# Evaluating the Performance of Ball-Milled Silk Fibroin Films for Simultaneous Adsorption of Eight Pharmaceuticals from Water

**DOI:** 10.3390/ijerph192214922

**Published:** 2022-11-13

**Authors:** Hlobsile Kgomo, Simiso Dube, Mathew Muzi Nindi

**Affiliations:** 1 Department of Chemistry, University of South Africa, Florida Campus, Cnr Christiaan de Wet Rd & Pioneer Avenue, Florida Park, Roodepoort 1709, South Africa; 2 Institute for Nanotechnology and Water Sustainability, College of Science Engineering and Technology, University of South Africa, Corner Christiaan de Wet and Pioneer Avenue, Florida Park, Roodepoort 1709, South Africa

**Keywords:** *A. mimosae*, silk, films, adsorption, pharmaceuticals

## Abstract

Pollutants mainly exist as multicomponent mixtures in the environment. Therefore, it is necessary to synthesize low-cost adsorbents that can simultaneously adsorb multiple compounds. This work presents the prospect of the adsorption of multiclass pharmaceuticals from the aqueous environment using an adsorbent derived from silk fibroin of the wild silkworm *Argema mimosae*. The adsorbent was prepared by dissolving degummed silk fibroin and the resultant solution was cast to obtain films that were ball-milled to powder. FTIR results revealed bands corresponding to N-H and C=O stretching vibrations. Particle size distribution data generally showed two size groups in the range of 50–90 nm and 250–625 nm. The study focused on the adsorptive removal of multiple compounds consisting of eight pharmaceuticals representing various classes including a β-blocker (pindolol), anesthetic (lidocaine), stimulant (caffeine), antiviral (nevirapine), steroid (estriol), anti-epileptic (carbamazepine), and a non-steroidal anti-inflammatory drug (naproxen). The adsorption process was best fitted to the pseudo-second-order isotherm and an overall match to the Freundlich model. Thermodynamic parameters suggested that the process was mainly exothermic and more spontaneous at lower temperatures. The performance of the adsorbent was further evaluated using environmental waters and the adsorbent demonstrated good potential for simultaneous adsorption of multicomponent pharmaceuticals.

## 1. Introduction

Pharmaceuticals and their by-products, personal care products, pesticides, and other pollutants of emerging concern (PECs) have been increasingly identified in the aquatic and terrestrial environment. Their pathways into the environment are mainly through wastewater treatment plant (WWTP) effluents and agricultural runoffs into surface water. The contamination of surface water by these pollutants of emerging concern poses a public health problem as the surface water is then recycled through water treatment plants that were not designed to eliminate PECs from the environment. Pharmaceutically active compounds (PhACs), including antibiotics, steroid hormones, antiretrovirals (ARVs), β-blockers, non-steroidal anti-inflammatory drugs (NSAIDs), and many others have been detected in wastewater effluents, albeit in trace amounts [1,2,3,4]. They are ingested as medication which may not be completely absorbed in the body, but a certain percentage undergoes structural changes producing metabolites. The lingering portion remains unaltered in the body and may be excreted in the parent form, metabolites, or conjugates through urine and/or fecal matter into wastewater sewage and/or directly into waters or soils [5]. 

Various PhACs have been detected in the water received by WWTPs, effluents, and also in sludge. They have also been detected in surface waters in South Africa, Kenya, China, and other countries [4,6,7]. Even though they were found in trace amounts, their intensive and continued use, the increased toxicity of their by-products, and their biotransformation along the treatment plant train increase their potential negative public health impacts [8]. Since conventional technologies for water treatment are inefficient for the complete removal of PhACs in water, various techniques have been explored including advanced oxidation processes (AOPs), advanced filtration techniques, and adsorption processes amongst others. These have generally shown better removal and/or degradation efficiencies of PhACs compared to conventional techniques [9,10]. Though effective, a shared concern among researchers is related to intermediates generated during the processes, some of which are persistent and more toxic than the parent compounds. For instance, acridine, an intermediate product formed during the photodegradation of carbamazepine, is reported to be mutagenic and carcinogenic [9,10]. Membrane filtration using nanofiltration (NF) and reverse osmosis (RO) membranes is another technique that has also been explored for water treatment, however, challenges associated with fouling and high operating costs limit their use [11].

Adsorption is usually the preferred method due to its efficiency, wider applicability, relatively low costs, and simplicity over other techniques, with limited generation of undesirable by-products [12]. Natural bio-based materials have been mostly preferred over synthetic materials since they are biocompatible and biodegradable after use [13]. For example, different forms of adsorbent materials derived from chitosan, moringa, and *Mondia whitei* have been explored as potential adsorbents for the adsorption of selected PhACs [14,15,16]. Silk-based forms from the domesticated *Bombyx mori* silkworm have been investigated as potential low-cost adsorbents for the adsorption of pollutants. For instance, Xiao et al. [17] performed batch adsorption studies for the adsorption of dyes using ball-milled silk fibroin (SF) powder while Song and co-workers [18] used SF films as the adsorbent material. In some studies, silk was blended to improve its performance for the intended purpose. For example, silk foams prepared by blending with orange peel powder were used for the adsorption of methylene blue. It was found that blending increased the adsorption capacities five-fold [19]. Verma and Subbiah [20,21] explored the feasibility of incorporating silk sericin, a by-product of degumming silk cocoons, for membrane modification. The modified membrane was used for the removal of ibuprofen from aqueous solutions. 

With over 400 species of wild silk-producing moths in the world, approximately eight types have been successfully beneficiated through various applications. The fabrication of an adsorbent based on silk fibroin presents an opportunity to exploit its physical and surface characteristics to adsorb selected PhACs and remove them from environmental samples [22,23,24]. Silk is an attractive material to explore as a potential low-cost adsorbent due to its biocompatibility and biodegradability after use. The investigations into their adsorption characteristics, through analysis of adsorption isotherms and kinetics, serve as a good measure for comparative investigations with other naturally prepared adsorbents and polymers and would provide data for the techno-economic evaluation of these adsorbents for pilot-scaling and further applications. In this work, the prospect of utilizing wild silk fibroin of *Argema mimosae* as a candidate low-cost adsorbent material was investigated. This work presents our findings on the application of ball-milled silk fibroin films for the simultaneous adsorption of eight pharmaceutical compounds. To our knowledge, there are no reports in the literature reporting the utilization of silk fibroin-based forms for the simultaneous adsorption of multiclass pharmaceuticals from water.

## 2. Materials and Methods

### 2.1. Chemicals and Materials

The pharmaceuticals used in this study were pindolol, lidocaine, nevirapine, naproxen, caffeine, prednisolone, estriol, and carbamazepine and their physicochemical properties are listed in Table 1. All the compounds were supplied by Sigma Aldrich with >95% purity. Analytical grade sodium carbonate (Na_2_CO_3_ ≥ 99.0%), lithium bromide (LiBr (≥ 99.0%), HPLC grade formic acid (99%), acetonitrile, and methanol were purchased from Sigma-Aldrich (St Louis, MO, USA). All aqueous solutions were prepared using ultrapure water (18.2 mΩ) produced through a Milli-Q- water purification system (Molsheim, France). The *A. mimosae* silkworm cocoons were collected from the Kingdom of Eswatini (former Swaziland) in Sithobelweni area under the Lubombo region.

### 2.2. Preparation of Silk Adsorbents

*A. mimosae* cocoons were degummed in a two-step degumming process for 90 min at each time as described in [23]. The cocoons were firstly boiled in 0.025 mol dm^−3^ then in 0.01 mol dm^−3^ Na_2_CO_3_ aqueous solutions. After each boiling step, the fibroin was washed with warm water and thereafter air-dried until constant weight. The degummed silk fibroin was then dissolved in a 5% LiBr—formic acid (FA) solution at 60 °C. The solution was cast on polystyrene Petri dishes and kept open under a fume hood until dry. The resultant films were immersed in deionized water to wash off any residual salt and FA and then air-dried. The resultant films were ball-milled using 12 mm stainless steel milling balls at a frequency of 25 Hz (approx. 1 500 min^−1^) for 90 min to obtain the powder adsorbent ready for application.

### 2.3. Characterization Techniques

The surface morphology of the silk adsorbent was investigated using a JEOL-JSM-6010 Plus/LA scanning electron microscope (Tokyo, Japan). The powder sample was fixed onto a stub using carbon tape and sputter-coated with a gold layer before analysis. Functional group analysis was carried out on a Vertex 70 version Fourier Transforms Infrared with Attenuated Total Reflection (FTIR-ATR) mode using OPUS 7.5 software (Bruker Optik GmbH, Ettlingen, Germany). 

### 2.4. Sampling and Sample Preparation

All samples were collected within the Gauteng Province of the Republic of South Africa. Influent and effluent samples were collected from the Daspoort Wastewater Treatment Plant in Pretoria which receives raw wastewater water from around the central Pretoria area. When the treatment is over, the treated water is discharged into the Apies River. Therefore, samples were also collected from the river. The samples were collected using the grab-sampling method and transported on ice to the laboratory. The samples were passed through glass wool and kept at −5 °C until use.

### 2.5. Batch Adsorption Experiments

Batch experiments were employed to investigate the adsorption characteristics and adsorption mechanisms of the pharmaceutical drugs on the silk fibroin film powder. Generally, in a batch adsorption method, a known quantity of adsorbent is mixed all at once with a known quantity of a solution and the system is agitated for a predetermined time. The resultant solution is separated by filtering, centrifuging, or decanting [25]. In this work, 10 mg (0.01 g) of the adsorbent was weighed into reaction vials, thereafter, a 15 mL (0.015 L) solution of mixed drugs was added. The experiments were carried out in a shaking water bath at an agitation speed of 100 rpm. The concentration of the drugs was 500 μg L^−1^ for lidocaine, pindolol, and estriol and 250 μg L^−1^ for the rest. The solution pH was adjusted using 0.01 mol L^−1^ of either HCl or NaOH to reach pH values in the range of 2–12. The effect of time on the adsorption of the different drugs was carried out from 2 to 180 min. To understand the effect of temperature and further determine the nature and feasibility of the adsorption of the pharmaceuticals onto the silk fiber, the adsorption experiments were carried out at 15, 25, and 45 °C (288, 298, and 318 K). Upon completion of each adsorption experiment, about 1 mL aliquot was filtered through a PVDF syringe filter with 0.45 µm pore size. The residual concentration of each analyte was determined using a high-pressure liquid chromatograph equipped with a diode array detector (HPLC-DAD). 

The adsorption capacity at equilibrium, *q_e_*, in µg g^−1^ was calculated using Equation (1):

(1) $$q_{e}=\frac{V(C_{i}-C_{e)}}{m}$$

where $C_{i}$ is the initial concentration of each analyte expressed in µg L^−1^; $C_{e}$ is the concentration of each analyte at equilibrium expressed in µg L^−1^; *V* is the volume of the solution in L; and *m* is the mass of the adsorbent in g. Therefore, the total adsorption capacity *Q_e_* (µg g^−1^) at equilibrium is:

(2) $$Q_{e}=\sum q_{e}$$

Adsorption kinetics were analyzed to evaluate the relationship between contact time and the adsorption of the pharmaceuticals. This was to mathematically determine the reaction rates and further elucidate the reaction mechanism driving the adsorption process. This was executed by fitting the experimental data to the pseudo-first-order (Equation (3)) and pseudo-second-order kinetic models (Equation (4)):

(3) $$\log\;\left(q_{e}-q_{t}\right)=\log q_{e}-\frac{K_{1}}{2.303}t$$

(4) $$\frac{t}{q_{t}}=\frac{1}{K_{2}q_{e}^{2}}+\frac{1}{q_{e}}t$$

where $q_{e}\;\mathrm{and}\;q_{t}$ (µg g^−1^) are adsorption at equilibrium and time *t*, respectively; $K_{1}$ (min^−1^) and $K_{2}$ (µg g^−1^ min^−1^) are the adsorption rate constants.

Using Equation (4), it is possible to estimate the initial adsorption rate $v_{o}$ (Equation (5)) and the time it takes to reach half of the maximum adsorption capacity *t*_1/2_ (Equation (6)):

(5) $$v_{o}=K_{2}q_{e}^{2}$$

(6) $$t_{1/2}=\frac{1}{K_{2}q_{e}^{2}}$$

The linearized forms of the Langmuir, Freundlich, and Temkin isotherm models described by Equations (7)–(9), respectively, were applied to evaluate the adsorption isotherms:

(7) $$\frac{1}{q_{e}}=\frac{1}{K_{L}q_{max}}\frac{1}{C_{e}}+\frac{1}{q_{max}}$$

(8) $$logq_{e}=\log K_{F}+\frac{1}{n}logC_{e}\;$$

(9) $$q_{e}=\frac{RT}{b_{T}}lnK_{T}+\frac{RT}{b_{T}}lnC_{e}$$

where $q_{e}$ is the amount of adsorbate adsorbed at the equilibrium concentration in µg g^−1^; $q_{max}\;$is the maximum adsorption capacity expressed in µg g^−1^; $C_{e}$ is the equilibrium concentration of the adsorbate in µg L^−1^; and $K_{L}$ is the Langmuir constant in L µg^−1^. $K_{F}$ is the Freundlich constant describing the affinity of the adsorbate for the active sites in µg^1−1/n^ L^−1^/^n^ g^−1^ and 1/n is a factor that describes the intensity of the adsorptive interactions. R is the ideal gas constant (8.314 J mol^−1^ K^−1^), T (K) is the absolute temperature,$\;K_{T}$ (L g^−1^) is the Temkin isotherm equilibrium binding constant and $b_{T}$ (J mol^−1^) is the Temkin isotherm constant. 

### 2.6. Application to Real Water Samples

The adsorption experiments were carried out using real water samples to investigate the effect of a complicated matrix on the adsorption of the selected pharmaceuticals. The water samples were spiked with a mixed standard solution to obtain a final concentration between 250 µg L^−1^ and 500 µg L^−1^. 

## 3. Results

### 3.1. Structure and Morphology of Silk Adsorbents

**Figure 1:**
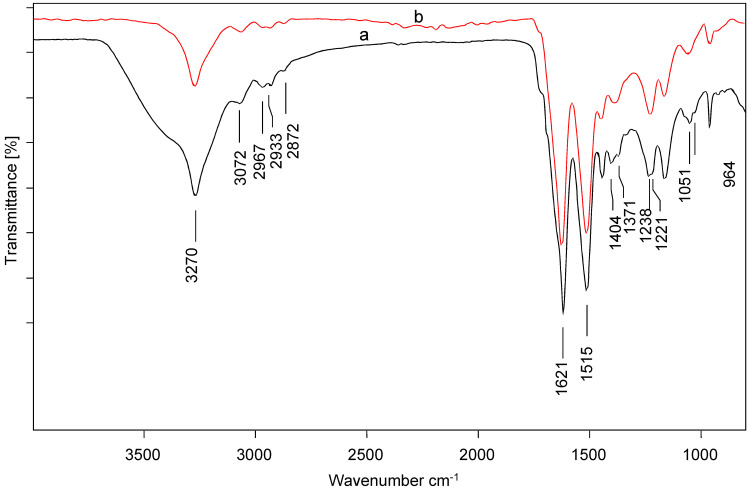
FTIR spectra of silk powder (a) before (b) after adsorption of pharmaceuticals.

The FTIR results of the silk powder before adsorption of the pharmaceuticals, respectively, are presented in Figure 1a. A broad band was observed between 3687–3105 cm^−1^ with a distinct peak around 3272 cm^−1^ attributed to N-H stretching. The broadness of the band suggested the presence of another functional group likely due to O-H stretching arising from the presence of water molecules. A strong amide I absorption band was observed at around 1621 cm^−1^ due to C=O stretching, and an amide II band at around 1515 cm^−1^ due to N-H bending and C-H stretching vibrations. Both bands were indicative of β-sheet molecular conformation. The band around 1404 cm^−1^ was attributed to C-O and O-H bending vibrations [26]. This was followed by a shoulder peak at around 1371 cm^−1^ which was assigned to C-H bending resulting from poly (glycine-alanine) sequences. Amide III twin bands were observed at around 1238 cm^−1^ arising from a combination of C-N stretching coupled with N-H bending vibrations and at 1221 cm^−1^ due to C-H bending combined with C-N stretching coupled with N-H bending. The band at around 1167 cm^−1^ was attributed to C-N stretching while the band at around 1051 cm^−1^ was assigned to C-OH and C-O stretching vibrations attributable to the presence of poly(glycine-alanine) amino acid sequences. A shoulder peak was observed around 1032 cm^−1^ assigned to random coils due to C-H rocking. A band attributable to poly(alanine) residues was observed at 964 cm^−1^ and N-H rocking vibrations, respectively, arising [27]. 

After the adsorption of the pharmaceuticals (Figure 1b), the characteristic bands of the silk powder were observed although some shifts were observed. For instance, no noticeable disruptions were observed for the bands attributed to β-sheets in the amide I and II regions. On the other hand, instead of the band at 1404 cm^−1^ and 1371 cm^−1^, a new band was observed at around 1335 cm^−1^. The twin bands observed at 1238 cm^−1^ and 1222 cm^−1^ were replaced by a single band around 1228 cm^−1^. Additionally, the bands at 1051 cm^−1^ and 1032 cm^−1^ were also disrupted resulting in a band at 1059 cm^−1^. This is an indication that the silk powder interacted with the pharmaceutical drugs likely forming hydrogen bonds.

**Figure 2:**
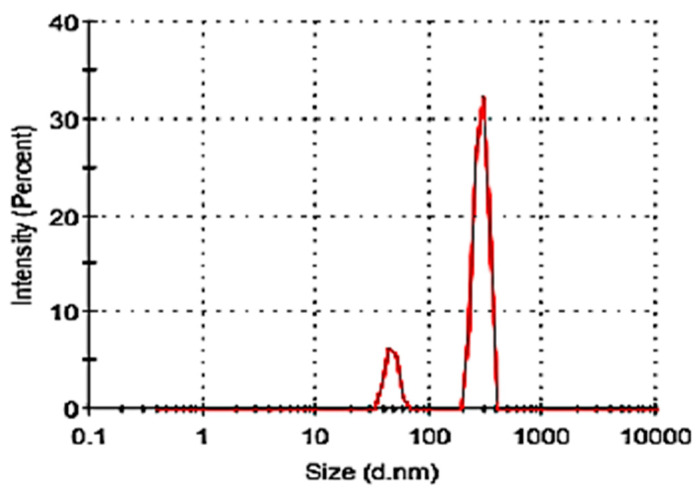
Particle size distribution of silk film powder.

The dynamic light scattering (DLS) measurements for the particle size distribution of the adsorbent, as shown in Figure 2, show a small particle size group in the range of 50 to 90 nm and a large particle size group that ranged from 250 to 625 nm. The polydispersity index was above 0.6 indicating that the adsorbent constituted multiple particle size populations, including large aggregates. However, the particle size was considerably smaller than the diameter of the degummed silk fibroin fibers which range between 22 and 48 μm. It is reported that to obtain silk powder with a particle size between 2–3 μm requires long milling times, and harsh pre-treatment processes must be employed in addition to the long milling times of up to 40 hrs [28]. While the powder still showed large aggregates, in the current study, however, the preparation of the silk films was carried out under relatively mild conditions to preserve the integrity of the silk fibroin with a milling time of 90 min [23].

**Figure 3:**
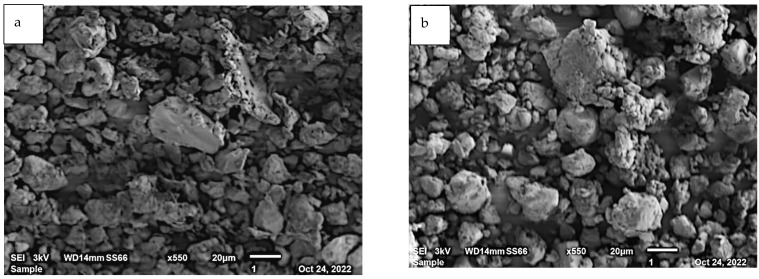
SEM images of silk film powder (**a**) before and (**b**) after adsorption of pharmaceuticals.

The SEM micrographs of the adsorbent before and after the adsorption of pharmaceuticals are shown in Figure 3a,b, respectively. The images reveal that before the adsorption process, the silk film powder consisted of irregularly shaped particles. After the adsorption of the pharmaceuticals, the particles seem to have assumed a more spherical shape with some aggregates. Previous studies have also found that silk particles produced by ball-milling tend to agglomerate via interparticle attraction to form fibrous aggregates [29].

### 3.2. Effect of pH

**Figure 4:**
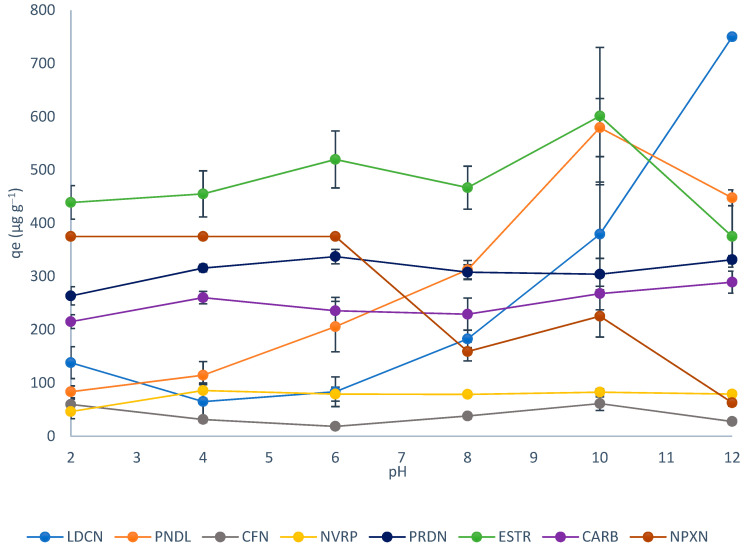
Effect of pH on the adsorption of pharmaceutical drugs.

The results of the effect of pH on the adsorption of the selected pharmaceuticals are presented in Figure 4. Under strongly acidic conditions (pH 2) the adsorption of pindolol, caffeine, and nevirapine was < 100 µg g^−1^, 200 µg g^−1^ < carbamazepine, prednisolone < 300 µg g^−1^, 300 µg g^−1^ < naproxen, and estriol < 500 µg g^−1^. Increasing the pH of the solution to slightly acidic (pH 4) did not affect the adsorption of naproxen while some improvements were observed in the adsorption of estriol, pindolol, prednisolone, carbamazepine, and nevirapine. A slight decline in the adsorption of lidocaine and caffeine was also observed. Under weak acidic conditions (pH 6), there was a noticeable increase in the adsorption of pindolol while no major changes were observed for the rest of the drugs. When the solution was adjusted to slightly basic (pH 8), a sudden drop in the adsorption of naproxen took place, while the adsorption of pindolol and lidocaine increased. The two continued to increase as the solution became a more basic solution (pH 10). A further increase in the solution’s pH to strongly basic (pH 12) resulted in a sharp decrease in the adsorption of estriol, naproxen, and pindolol. On the other hand, a great improvement was observed for lidocaine.

### 3.3. Adsorption Kinetics

**Figure 5:**
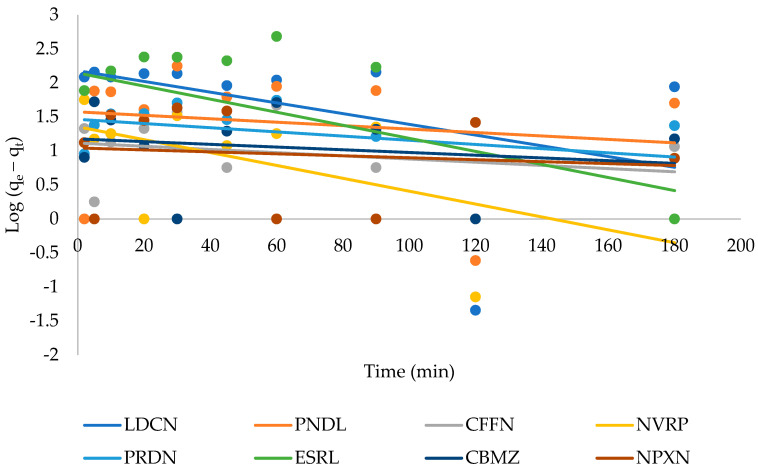
Pseudo-first-order plot for the adsorption of pharmaceutical drugs.

**Figure 6:**
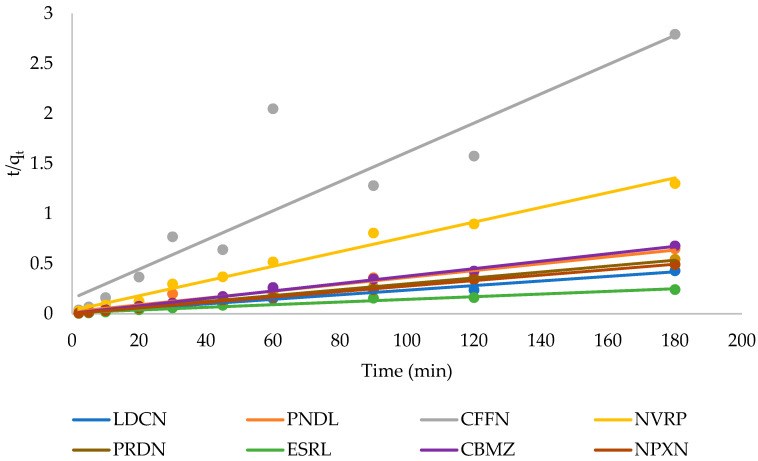
Pseudo-second-order plot for the adsorption of pharmaceuticals.

The adsorption kinetics were investigated by fitting the experimental data to the pseudo-first-order (Equation (3)) and pseudo-second-order models (Equation (4)), the linear plots are given in Figure 5 and Figure 6, respectively, while the kinetic parameters are listed in Table 2. The linear regression coefficients for the pseudo-first-order model were very low for all the pharmaceuticals; moreover, a wide difference was observed between the calculated $q_{e\;cal}$ values and the experimentally determined $q_{e\;exp}$ values. The pseudo-second-order model showed better linearity confirming its suitability for describing the adsorption process. This suggests that chemisorption was the rate-limiting step in the adsorption of the pharmaceuticals [30,31]. In addition, the experimentally determined values of *q*_e_ and the calculated values were also close. The *t*_1/2_ values revealed fast kinetic adsorption processes in the range of 1.00 to 10.40 min. In addition, naproxen showed the highest initial adsorption rate of 370 μg g^−1^ min^−1^, while caffeine had the slowest rate of 7 μg g^−1^ min^−1^.

### 3.4. Adsorption Isotherms

The ability of the silk powder to adsorb the pharmaceuticals was further investigated by determining the maximum adsorption for each of the pharmaceuticals according to the Langmuir model and the adsorption isotherm parameters listed in Table 3. The largest $q_{max}$ of 5000 μg g^−1^ was obtained for estriol and carbamazepine while caffeine had the lowest value of 40 μg g^−1^. However, the model showed poor linearity for some of the compounds such as lidocaine with R^2^ of 0.503 indicating a poor fit to the isotherm. On the other hand, the data showed a better fit to the Freundlich model, suggesting that the adsorption process took place on a heterogeneous surface. The values of 1/n were in the range of 0.73–2.0, where pindolol, nevirapine, carbamazepine, and estriol achieved 1/n < 1, suggesting normal adsorption while the rest of the compounds had 1/n > 1 indicative of a Freundlich cooperative multi-layer adsorption mechanism. Except for lidocaine and caffeine, the pharmaceuticals showed $K_{F}\;$≥ 1 suggesting a good affinity for the active sites of the adsorbent. The highest $K_{F}$ value was obtained for estriol indicating a stronger affinity for the available active sites. The lowest $K_{F}$ value of 1.16 × 10^−4^ was obtained for lidocaine yet an appreciable $q_{max}$ value was obtained compared to caffeine. Such behavior could likely be due to the positive cooperative nature of the adsorption (described by 1/n > 1) which allows other compounds in the mixture to enhance the adsorption of low-affinity compounds such that they are adsorbed to a greater extent [32]. The experimental data for several compounds showed good linearity for both the Freundlich and Langmuir isotherms proposing that both isotherms could be applicable. This suggests that the adsorbate monolayer may have been formed first and the heterogeneous multi-layers followed thereafter [33]. This may have occurred as a result of solute–solute attractive forces between the adsorbate on the surface of the adsorbent and the adsorbate in the solution. Finally, the experimental data were fitted to the Temkin model. According to the model, $b_{T}$ > 0 indicates that the adsorption processes were exothermic [34].

### 3.5. Thermodynamic Studies

Batch adsorption studies were carried out at different temperatures (288, 298, and 318 K) to determine the nature and feasibility of the adsorption. This was executed by determining the standard Gibbs free energy change (ΔG°, kJ mol^−1^), standard enthalpy change (ΔH°, kJ mol^−1^), and standard entropy change (ΔS°, J mol^−1^ K^−1^) values for the adsorption process through the following equations:

(10) $$\Delta G=-RT\;Ln\;K_{c}$$

(11) $$K_{c}=\frac{q_{e}}{C_{e}}$$

(12) ΔG°=ΔH°−TΔS°

Therefore:

(13) $$Ln\;K_{c}=\frac{\Delta S{}^{\circ}}{R}-\frac{\Delta H{}^{\circ}}{RT}$$

where R is the ideal gas constant ((8.314 J mol^−1^K^−1^) and T (K) is the temperature. 

The plot of $Ln\;K_{c}$ vs. 1/T was used to determine the values of ΔH° and ΔS° from the slope and coefficient, respectively. The corresponding thermodynamic parameters are given in Table 4. The positive ΔG° values for caffeine and nevirapine were an indication of the non-spontaneous adsorption of the pharmaceuticals. On the other hand, ΔG° for lidocaine and pindolol was negative at lower temperatures and became positive when the temperature increased to 318 K (45 °C). This was an indication that the adsorption of the pharmaceuticals was spontaneous at lower temperatures. The rest of the pharmaceuticals, on the other hand, showed negative ΔG° for the temperatures investigated. However, the values increased with increasing temperatures indicating that higher temperatures resulted in a decrease in the spontaneity of the adsorption process. This implies that the sorption process was less favorable at higher temperatures. An adsorption process may either be exothermic or endothermic which occurs when ΔH° < 0 and ΔH° > 0, respectively. Therefore, except for naproxen, the values of ΔH° were negative, indicating an exothermic nature of the sorption. Additionally, the negative ΔS° values were reflecting a decrease in randomness on the interface during the sorption process suggesting that the process was enthalpy driven [35]. These results suggest that hydrogen and van der Waal’s were the dominant driving forces of the adsorption process. However, the positive values of ΔH° and ΔS° obtained for naproxen are suggestive of an endothermic mechanism driven by hydrophobic interactions between the aromatic rings of naproxen and the silk surface [20,36].

### 3.6. Adsorption Mechanisms

Generally, a combination of factors including solubility, pK_a_, log K_ow_, functional groups, and structure can influence the adsorption of pharmaceutical compounds. For instance, the solubility of naproxen was reported to be pH-dependent and shows appreciable solubility as pH increases [37]. Therefore, around pH < 2 the adsorption of naproxen was likely due to its poor solubility in water. Around pH 4, naproxen is expected to be neutral while silk exists in its zwitterionic form, therefore, non-electrostatic interactions such as hydrogen bonding would be expected to contribute to the adsorption. Since the pK_a_ of naproxen is about 4.2, it is expected that under basic conditions naproxen will exist in its ionic form while the silk surface also becomes densely populated with negative charges. Hence, charge repulsions are likely to take place which would result in poor adsorption. This would explain the sharp decrease in the adsorption of naproxen as observed in Figure 3. Similar observations have also been observed elsewhere [38].

Pindolol is a weak base with an amine group that can be protonated when pH < pk_a_ (9.25) making the cationic form of pindolol the dominant species. Since silk is also protonated around pH 2, charged repulsion with the cationic form of pindolol resulted in its poor adsorption. However, around pH 4–5, silk exists in its zwitterionic form which would allow weak electrostatic attractions to take place resulting in slight improvements as observed around pH 4. With increasing pH, the silk surface becomes densely populated with negative charges which would result in strong electrostatic attractions with the cationic form of pindolol and thus high adsorption. After pH 10, an abrupt drop in the adsorption of pindolol was observed which may be associated with the deprotonation of pindolol producing a negatively charged species. Therefore, charge repulsion between the anionic form of pindolol and silk, which also bears a net negative charge under the basic conditions, affected the adsorption. A similar trend was observed for the adsorption of lidocaine since it also has a weakly basic amine group which can be protonated when pH < pK_a_ (7.86).

Estriol exists predominantly in its non-ionic state when pH < pK_a_ (10.4) therefore, interactions between the adsorbent and estriol were likely due to H-bonding. However, under strongly basic conditions (pH 12), the phenolic group of the estriol forms an anionic species [39,40]. Since silk also bears a negative charge under basic conditions it promotes electrostatic charge repulsion causing a sharp decline in the adsorption efficiency. Carbamazepine is a neutral drug that depends on adsorption via H-bonding. Similarly, prednisolone exists in its neutral form between pH 2.9–12.59; therefore, in the pH range investigated, the adsorption was likely due to H-bonding. On the other hand, the recalcitrant behavior of caffeine to pH variations may be due to its hydrophilic nature with log *K*_ow_ −0.07 coupled with high water solubility of about 21 600 mg L^−1^. Therefore, poor adsorption of caffeine was observed. With nevirapine, however, the relatively low adsorption almost throughout the entire pH range investigated might be associated with its pK_a_ of 2.8. Under extremely acidic conditions (pH 2), the low adsorption might be due to competitive sorption for the active sites with the H^+^ ions [30]. However, when the solution pH > pK_a_, nevirapine exists in its anionic form, and this was accompanied by an increase in the adsorption likely associated with electrostatic attractions due to the presence of positive charges on the zwitterionic silk [41]. A further increase in the pH results in a negatively charged silk surface causing a slight decline in the adsorption of the drug. Consequently, the adsorption remains nearly constant for the rest of the pH conditions.

### 3.7. Performance of Silk Adsorbent in Environmental Water Samples

**Figure 7:**
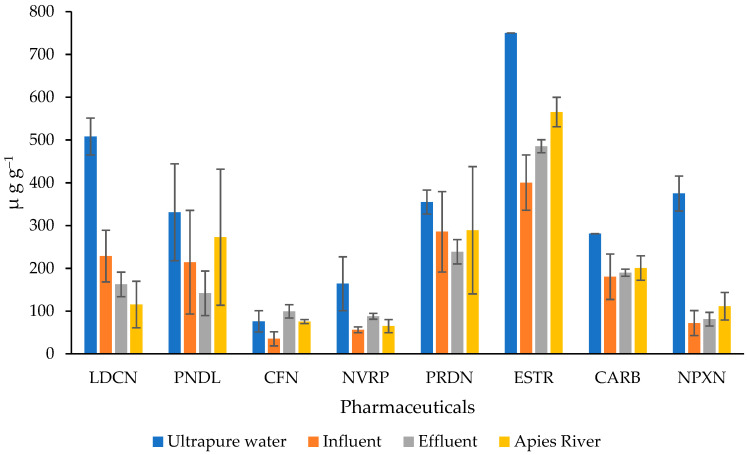
Adsorption of selected pharmaceuticals in environmental water.

The results of the adsorption of the selected pharmaceuticals on the silk adsorbent are depicted in Figure 7. Generally, the adsorption capacities of the eight pharmaceuticals were lower in real samples compared to the ultra-pure water. Such a trend is not unusual since environmental samples present a more complex matrix containing organic and inorganic pollutants which may compete with the compounds of interest for the active sites of the adsorbent material and thus hinder the adsorption of the target analytes. The highest adsorption was observed for estriol in all the water types. This is in accordance with the results reported for the adsorption isotherms in which estriol showed the highest affinity for the active sites of the silk adsorbent. The total adsorption capacities *Q_e_* described by Equation (2), were found to be 2840, 1695, 1486, and 1473 µg g^−1^ for ultra-pure water, Apies River, effluent, and influent water samples, respectively.

The performance of the silk adsorbent was compared with other adsorbents reported in the literature (Table 5). Although the adsorption capacities achieved by the silk adsorbent in the current study were relatively low, the adsorbent has however demonstrated good potential for the adsorption of multi-class pharmaceuticals even in complicated matrices such as influent and effluent water. For instance, Kebede et al. [31] used nanofibers derived from a blend of moringa seed protein and polyvinyl alcohol (PVA) for the adsorption of selected NSAIDs and carbamazepine. They reported an adsorption capacity $q_{e\;exp}$ of 0.0353 mg g^−1^ (35.3 µg g) for carbamazepine which was lower than that demonstrated by the silk adsorbent in the current study. Additionally, higher removal efficiencies were achieved for carbamazepine compared to powdered activated carbons derived from coal, peat, and coconut shells [41]. On the other hand, the other adsorbents showed superior performance to the silk adsorbent evidenced by the high adsorption capacities that were achieved.

## 4. Conclusions

The performance of silk film powder as an adsorbent for the simultaneous adsorption of multiclass pharmaceuticals from water was demonstrated. The target pharmaceuticals included a β-blocker, anesthetic, stimulant, antiviral, anti-epileptic, NSAID, and steroids. The adsorbent was derived from an African wild silk fibroin of *A. mimosae* silkworm. A simple approach of solubilization of silk fibroin and casting to obtain silk films that were ball-milled to a powder was used. Its ability to adsorb the pharmaceuticals was investigated under different conditions such as pH, contact time, and temperature. The pH of the solution was found to greatly influence the adsorption of the pharmaceuticals. The maximum adsorption capacities ($q_{max}$ ) predicted by the Langmuir isotherm were in the range of 40 to 5000 μg g^−1^, with caffeine showing the lowest and both estriol and carbamazepine the highest. However, the overall adsorption kinetics were best described by the pseudo-second-order model indicating chemisorption as the rate-limiting step. Some of the pharmaceuticals such as estriol matched both the Freundlich and Langmuir isotherms likely due to solute–solute interactions, but the Freundlich was the best overall fit, while the Temkin model predicted that the adsorption was exothermic. The thermodynamics also showed that apart from naproxen, the adsorption process was exothermic in nature indicated by the negative ΔH° values, suggesting the process to be an energy sustainable approach. The adsorption of the pharmaceuticals proceeded mainly via interactive mechanisms which included van der Waals forces, electrostatic interactions, and hydrogen bonding. FTIR spectra confirmed the interaction of the fiber with the pharmaceuticals by the appearance of new bands while others disappeared. The total adsorption capacities were found to be 2840, 1695, 1486, and 1473 µg g^−1^ for ultra-pure water, Apies River, effluent, and influent water samples, respectively. While the adsorption capacity of the adsorbent was low compared to other adsorbents that have been used for similar work, the silk adsorbent has the potential to simultaneously adsorb pharmaceuticals even in complicated matrices such as influent and effluent. Further work should be carried out to investigate possible ways of improving the performance of the adsorbent possibly by surface modification and/or functionalizing.

## Figures and Tables

**Table 1:** Physicochemical properties and structures of selected pharmaceutical drugs.

Compound	Acronym	Class	CAS	Molecular Weight (g mol^−1^)	Log K_ow_	pKa	Structures
Pindolol	PNDL	β-blocker	13523-86-9	248.3	1.75	9.25	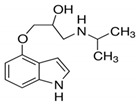
Lidocaine	LDCN	Anesthetic	137-58-6	234.3	2.26	7.86	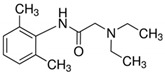
Caffeine	CFN	Stimulant	58-08-2	194.2	−0.07	10.4	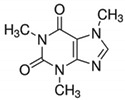
Nevirapine	NVRP	Antiviral	129618-40-2	266.3	3.89	2.8	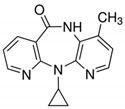
Prednisolone	PRDN	Steroid	50-24-8	360.4	1.62	2.912.59	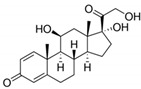
Estriol	ESTR	Steroid	50-27-1	288.4	2.45	10.4	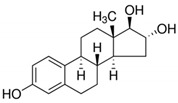
Carbamazepine	CARB	Anti-epileptic	298-46-4	236.3	2.45	13.95	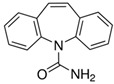
Naproxen	NPXN	NSAID	22204-53-1	230.2	3.18	4.15	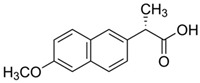

**Table 2:** Kinetic parameters of pseudo-first-order and pseudo-second-order.

		Pseudo-First-Order				Pseudo-Second-Order			
Pharmaceuticals	$q_{e\;exp}$ μg g^−1^	$q_{e\;cal}$ μg g^−1^	$K_{1}$ min^−1^	R^2^	$q_{e}$ μg g^−1^	$K_{2}$ min^−1^	$t_{1/2}$ min	$v_{o}$ μg g^−1^ min^−1^	R^2^
Lidocaine	508	151	1.8 × 10^−2^	0.179	435	8.4 × 10^−4^	2.7	158	0.979
Pindolol	331	38	5.8 × 10^−3^	0.024	294	5.7 × 10^−4^	6.0	49	0.965
Caffeine	76	13	5.4 × 10^3^	0.062	68	1.4 × 10^−3^	10.4	7	0.832
Nevirapine	164	23	2.2 × 10^−2^	0.367	135	1.7 × 10^−3^	4.4	31	0.985
Prednisolone	355	29	7.1 × 10^−3^	0.123	333	2.3 × 10^−3^	1.3	250	0.996
Estriol	750	140	2.2 × 10^−2^	0.246	769	1.3 × 10^−4^	10.4	74	0.962
Carbamazepine	281	15	4.6 × 10^−3^	0.036	270	3.7 × 10^−3^	1.1	238	0.996
Naproxen	375	11	3.3 × 10^−3^	0.014	370	2.7 × 10^−3^	1.0	370	0.998

**Table 3:** Adsorption isotherm parameters.

		Langmuir			Freundlich			Temkin	
Pharmaceutical	$q_{max}$ μg g^−1^	$K_{L}$ L μg^−1^	R^2^	1/n	$K_{F}$	R^2^	$b_{T}$ J mol^−1^	$K_{T}$ L μg^−1^	R^2^
Lidocaine	400	2.3 × 10^−3^	0.503	2.0	1.2 × 10^−4^	0.985	8.0	2.1 × 10^−3^	0.958
Pindolol	556	4.5 × 10^−4^	0.992	0.73	1.6	0.998	20.4	4.5 × 10^−3^	0.965
Caffeine	40	4.2 × 10^−3^	0.934	0.43	2.9 × 10^−3^	0.908	16.9	154	0.934
Nevirapine	769	8.9 × 10^−4^	0.991	0.71	1.2	0.989	17.8	51	0.937
Prednisolone	500	3.9 × 10^−2^	0.751	1.3	1.0	0.976	3.4	1.6 × 10^−2^	0.883
Estriol	5000	1.2 × 10^−3^	0.994	0.89	8.9	0.998	2.2	1.4 × 10^−2^	0.996
Carbamazepine	5000	4.0 × 10^−4^	0.983	0.99	2.4	0.988	5.3	1.3 × 10^−2^	0.922
Naproxen	1429	2.0 × 10^−3^	0.880	1.54	5.0	0.908	2.4	2.5 × 10^−1^	0.955

**Table 4:** Thermodynamic parameters.

Phamaceuticals	R^2^	ΔG°			ΔH° (KJ mol^−1^)	ΔS° (KJ mol^−1^)
		288 K	298 K	318 K		
Lidocaine	0.926	−3.5	−1.1	0.8	−42.9	−0.1
Pindolol	0.914	−4.1	−3.9	2.2	−68.9	−0.2
Caffeine	0.991	2.1	3.8	6.3	−37.4	−0.1
Nevirapine	0.995	0.48	2.4	7.5	−68.0	−0.2
Prednisolone	0.995	−4.4	−4.3	−4.1	−7.4	−0.01
Estriol	0.957	−3.9	−3.4	−3.0	−12.3	−0.03
Carbamazepine	0.938	−3.2	−1.2	−1.1	−24.6	−0.07
Naproxen	0.933	−3.2	−1.3	−1.1	97.2	0.3

**Table 5:** Removal efficiencies or adsorption capacities of selected pharmaceuticals with various adsorbents^−1^.

Pharmaceuticals	Adsorbent	Water Type	Adsorbent Dose	Removal Efficiency/Adsorption Capacity	Ref
**Carbamazepine**	MIP	Dam water	10 mg L^−1^	418 ng mg^−1^	[30]
	*Moringa* protein /PVA nanofibersseed	Ultrapure water	10 mg	0.0353 mg g^−1^	[31]
	Clay	Ultrapure water	25 mg	32 mg g^−1^	[42]
	PAC (wood)	Wastewater	10 mg/L	63%	[43]
	PAC (Coal)	Wastewater		44%	
	PAC (Peat)	Wastewater		48%	
	PAC (Coconut)	Wastewater		16%	
	Silk film powder	River water	10 mg	48%	Current study
		Wastewater inf		50%	
		Wastewater eff		53%	
**Nevirapine**	MIP	Dam water	10 mg	299 ng mg^−1^	[30]
	*Mondia whitei/*PVA nanofibers	Wastewater Inf	40 mg	174 mg g^−1^	[15]
		Wastewater eff		189 mg g^−1^	
		Deionized water		201 mg g^−1^	
**Naproxen**	Clay	Ultrapure water	25 mg	37 mg g^−1^	[42]
**Lidocaine**	*Mondia whitei/*PVA nanofibers	Wastewater Inf	40 mg	61 mg g^−1^	[15]
		Wastewater eff		5 mg g^−1^	
		Deionized water		73 mg g^−1^	
**Prednisolone**	*Mondia whitei/*PVA nanofibers	Wastewater Inf	40 mg	113 mg g^−1^	[15]
		Wastewater eff		154 mg g^−1^	
		Deionized water		174 mg g^−1^	

## Data Availability

The data presented in this study are available on request from the corresponding authors.
